# Identifying factors for pembrolizumab eligibility in head and neck cancer

**DOI:** 10.1007/s00432-025-06121-0

**Published:** 2025-02-07

**Authors:** Satoru Miyamaru, Daizo Murakami, Kohei Nishimoto, Yorihisa Orita

**Affiliations:** Department of Otolaryngology-Head and Neck Surgery, Graduate School of Medicine, Kumamoto University, 1-1-1, Honjo, Chuo-ku, Kumamoto 860-8556 Japan

**Keywords:** Pembrolizumab, Head and neck cancer, Immune checkpoint inhibitors, Combined positive score (CPS), Survival analysis

## Abstract

**Purpose:**

Although immune checkpoint inhibitors (ICIs) are used as first-line treatments for recurrent or metastatic head and neck cancer (R/M HNC), there are many cases where the treatment is ineffective, making the assessment of efficacy crucial. In this study, we examined factors associated with the therapeutic effects of pembrolizumab.

**Methods:**

We retrospectively analyzed 54 patients with R/M HNC treated with pembrolizumab from January 2020 to December 2022. We investigated the relationship between survival rates and various factors such as the combined positive score (CPS), histological subtypes, recurrent lesions, details of administered agents, sequence of administration, history of cetuximab use, and presence of immune-related adverse events (irAEs).

**Results:**

The overall survival rates at 1-, 2, and 3 years were 57.4%, 41.8%, and 32.3%, respectively. The response and disease control rates were 31.5% and 51.9%, respectively. In the univariate analysis, a CPS of 20 or higher, first-line treatment, no history of cetuximab use, and the presence of irAEs was associated with better survival rates, whereas in the multivariate analysis, the first two factors were significantly associated with better survival. In this study, 16 of 20 cases had a CPS of 50 or higher, and 7 had a CPS of 90 or higher, indicating that a large number of high CPS cases were included, which is believed to have contributed to the results of this study.

**Conclusion:**

In patients with a CPS of 20 or higher, pembrolizumab can be administered as first-line treatment, with favorable expected therapeutic effects.

## Introduction

Head and neck squamous cell carcinoma (HNSCC) is frequently diagnosed at advanced stages. Despite aggressive treatment, approximately 50% of patients with locally advanced HNSCC experience recurrence or metastasizes within two years of the initial treatment (Sacco et al. 2015). The prognosis for patients with recurrent or metastatic HNSCC (R/M HNSCC) used to be poor, with a median survival time of 4–5 months (Kowalski, Carvalho 2000). However, the introduction of cetuximab marked a turning point in R/M HNSCC treatment outcomes, significantly extending median overall survival in combination therapy to 10.1 months, compared to 7.4 months in the chemotherapy-only group (Vermorken et al. 2008). More recently, immune checkpoint inhibitors (ICIs) have demonstrated superior efficacy over cetuximab and are now considered the first-line treatment for R/M HNSCC. In a clinical trial, the 1-year and 2-year overall survival (OS) rates in the nivolumab treatment group for patients resistant to platinum-based agents were 36.0% (Ferris et al. 2016) and 16.9% (Yen et al. 2020), respectively, demonstrating its superiority over the investigator’s choice group, which included cetuximab. For patients without prior platinum agents, pembrolizumab monotherapy achieved a 2-year OS rate of 27%, whereas pembrolizumab combined with chemotherapy achieved a 2-year OS rate of 29%. These results demonstrate that pembrolizumab monotherapy and combination therapy are respectively non-inferior and superior to the cetuximab and chemotherapy combination (EXTREME regimen) (Vermorken et al. 2008; Burtness et al. 2019). Although these results indicate favorable outcomes relative to conventional treatments, the number of cases in which these treatments are effective is limited. Additionally, both drugs are costly, and chemotherapy has shown enhanced efficacy when administered after ICIs (Harrington et al. 2022; Saleh et al. 2019; Iwaki et al. 2024). Consequently, in cases where treatment efficacy is not expected, it is important to consider switching drugs early. Identifying predictive factors for treatment efficacy is, therefore, essential to inform decisions regarding ICI administration. While numerous studies have sought to identify indicators of treatment efficacy, no definitive consensus has been reached thus far, and further accumulation of clinical data is necessary. In this study, we retrospectively analyzed cases of R/M head and neck cancer (HNC) treated with pembrolizumab in our department to investigate the factors associated with treatment efficacy. Additionally, we analyzed the clinical course of patients who discontinued pembrolizumab for reasons other than tumor progression, despite initially achieving efficacy, to evaluate whether pembrolizumab treatment could be stopped, at what time, and in which cases it might be appropriate.

## Materials and methods

This retrospective study included 54 consecutive patients with recurrent or metastatic head and neck cancer (R/M HNC) who received pembrolizumab in our department between January 2020 and December 2022. Informed consent was obtained from all participants, and the study design was approved by the Institutional Review Board of Kumamoto University Hospital (Number 2338). This study was conducted in accordance with the principles of the Declaration of Helsinki.

In our department, surgery was considered the primary option for recurrences after curative treatment when feasible. However, if surgery is not feasible or is undesired by the patient, other treatment options are explored. Depending on the extent of the lesions and the patient’s overall condition, in addition to chemotherapy, we considered treatments such as radiation therapy, photoimmunotherapy (Cognetti et al. 2021; Tahara et al. 2021), or boron neutron capture therapy (Barth et al. 2012) by the Cancer Board at our institution. For chemotherapy, pembrolizumab was selected as the ICI for patients who are sensitive to platinum-based agents or had not used them before. For patients with a combined positive score (CPS) of 20 or higher, pembrolizumab monotherapy was the standard approach. For a CPS between 1 and 20, the choice between monotherapy and combination therapy with chemotherapy was determined basis of the patient’s overall condition and the progression rate of the disease. If the CPS was less than 1, a combination of pembrolizumab and chemotherapy or a regimen combining cetuximab and chemotherapy was selected according to the patient’s condition and disease status. If the patient was resistant to platinum-based agents, nivolumab or cetuximab plus chemotherapy was administered. Pembrolizumab monotherapy was administered for 3 weeks (200 mg) or 6 weeks (400 mg). In the combination therapy, pembrolizumab was administered with fluorouracil, cisplatin, or carboplatin for up to six cycles. The treatment response was evaluated through physical examinations and imaging approximately every three months. In cases with no immune-related adverse events (irAEs) necessitating suspension or discontinuation and where tumor size was shrinking or not increasing in size, pembrolizumab administration was continued. If the tumor increased in size, the Cancer Board discussed whether to continue or change the medication.

For all cases, we calculated the response rate (complete response [CR] + partial response [PR]) and disease control rate (CR + PR + stable disease [SD]) based on the best treatment response and the 1-year, 2-year, and 3-year OS rates. The content and number of cases related to irAEs were also examined. Factors potentially influencing survival were analyzed using both univariate and multivariate analyses. These factors included age, histological type (SCC vs. others), CPS (≥ 20 vs. 1 to less than 20), regimen administered (monotherapy vs. combination therapy), treatment sequence of pembrolizumab (first-line vs. second-line or later), history of cetuximab administration, presence or absence of irAEs, and recurrence site (single vs. multiple, local vs. distant). Single-site recurrence refers to metastasis that is limited to a single organ and includes multiple metastatic lesions within the same site. Additionally, we reviewed the subsequent clinical course of patients who discontinued pembrolizumab for reasons other than progressive disease (PD) despite initial tumor response. We also investigated the characteristics of patients who maintained response following pembrolizumab discontinuation.

OS was calculated using the Kaplan–Meier method, with differences in survival rates assessed for statistical significance using the log-rank test. Univariate and multivariate analyses were conducted with Cox proportional hazard models. All statistical analyses were performed using JMP 14 software (SAS Institute, Cary, NC, USA). Statistical significance was set at p < 0.05.

## Results

### Patient characteristics

Of the 54 patients with R/M HNC, there were 51 males and 3 females, with ages ranging from 28 to 85 years and a median age of 68 years. The observation period after pembrolizumab administration ranged from 1 to 47 months (median, 15 months). Among the 54 cases reviewed, the most common cancer type was hypopharyngeal cancer (21 cases), followed by oropharyngeal cancer (15 cases) and salivary duct cancer (8 cases). The most frequent histological type was squamous cell carcinoma (45 cases), followed by salivary duct carcinoma (four cases), mucoepidermoid carcinoma (two cases), adenoid cystic carcinoma, undifferentiated carcinoma, and carcinoma ex pleomorphic adenoma (one case each). The CPS was 20 or higher in 20 cases, between 1 and 20 in 31 cases, and none of the cases had a CPS < 1. Among the 20 cases with a CPS of 20 or higher, 16 had a CPS of 50 or higher, and 7 had a CPS of 90 or higher. In three cases, CPS was not measured because of the following reasons: the desire to initiate treatment as quickly as possible, the lack of appropriate specimens for testing, and the oversight of the attending physician. The administered regimen was pembrolizumab monotherapy in 45 patients and combination therapy in nine patients. In terms of treatment sequence, 43 patients received first-line therapy, and 11 patients received second-line therapy or later (Table 1).

**Table 1:** Patient characteristics

Characteristics		n = 54
Sex		
	Male	51 (94.4)
	Female	3 (5.6)
Age, Median [Min, Max] (years)		68 [28, 85]
Follow-up periods, Median [Min, Max] (months)		15 [1, 47]
Primary site		
	Hypopharynx	21 (38.9)
	Oropharynx	15 (27.8)
	Salivary gland	8 (14.8)
	Nasopharynx	3 (5.6)
	Sinonasal tract	3 (5.6)
	Oral cavity	2 (3.7)
	Larynx	2 (3.7)
Pathology		
	Squamous cell carcinoma	45 (83.3)
	Salivary duct carcinoma	4 (7.4)
	Mucoepidermoid carcinoma	2 (3.7)
	Adenoid cystic carcinoma	1 (1.9)
	SMARCB-1 deficient carcinoma	1 (1.9)
	Carcinoma ex pleomorphic adenoma	1 (1.9)
CPS		
	< 1	0
	1 ≤ < 20	31 (57.4)
	≥ 20	20 (37.0)
	Not measured	3 (5.6)
Treatment regimen		
	Monotherapy	45 (83.3)
	Combination therapy	9 (16.7)
Treatment line		
	1st	41 (75.9)
	≥ 2nd	13 (24.1)

*MIN* minimum, *MAX* maximum, *CPS* combined positive score

### Treatment efficacy

**Fig. 1:**
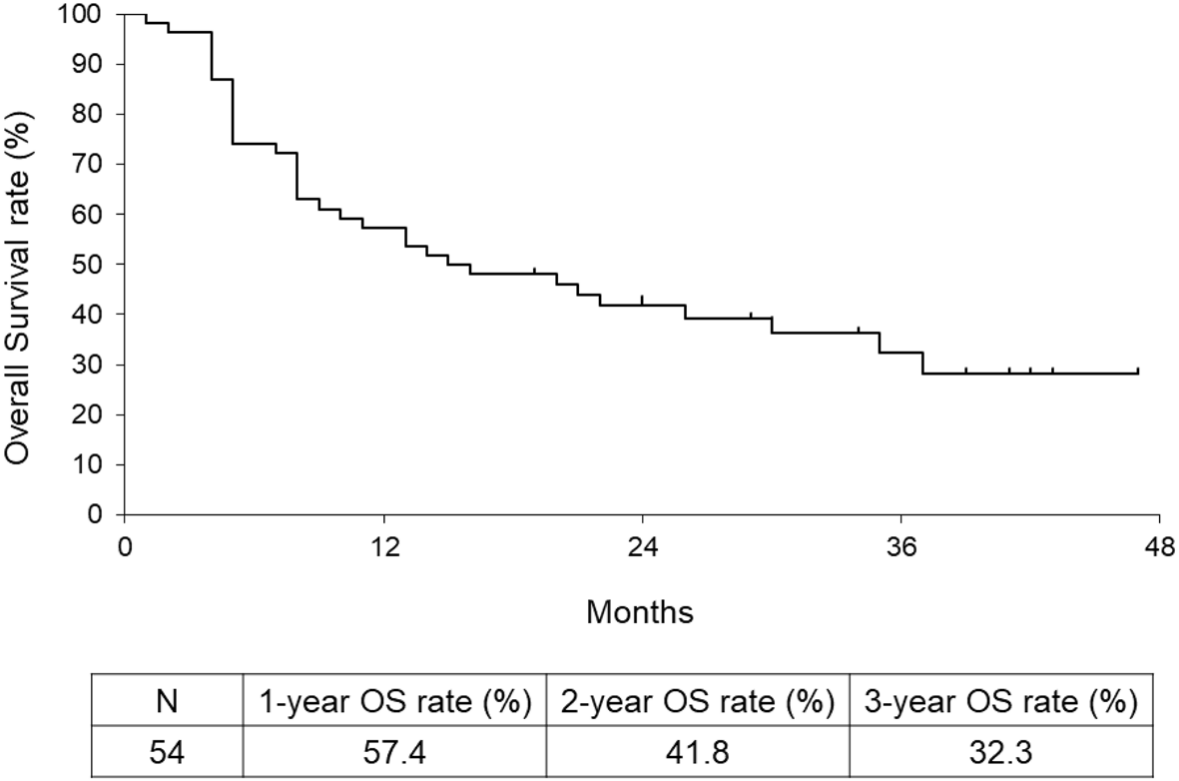
Overall survival curve for all 54 cases. OS: overall survival

Among 54 cases, there were 5 cases of CR, 12 cases of PR, 11 cases of SD, and 26 cases of PD. The overall response and disease control rates for all patients were 31.5% and 51.9%, respectively. Figure 1 shows the survival curves for all cases. The 1-year, 2-year, and 3-year OS rates were 57.4%, 41.8%, and 32.3%, respectively.

IrAEs were observed in 16 cases, including five cases of rash, four cases of hypothyroidism, two cases each of pneumonia, arthritis, and liver dysfunction, and one case of hypopituitarism. Table 2 shows the details of all 16 cases regarding the grade of irAEs, their onset timing, and subsequent outcomes.

**Table 2:**
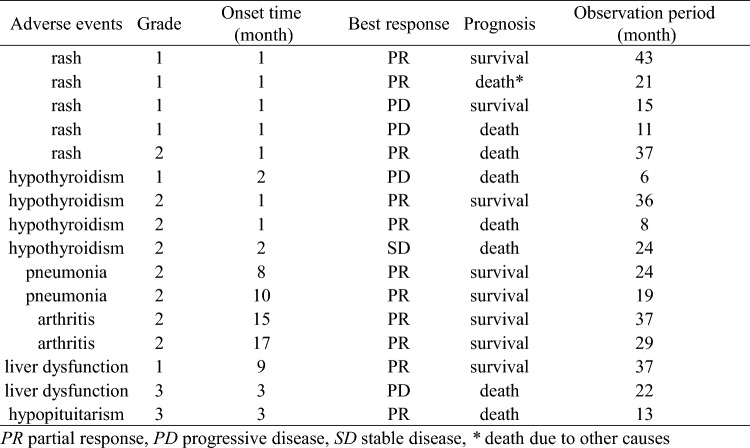
Details of immune –related adverse events

**Fig. 2:**
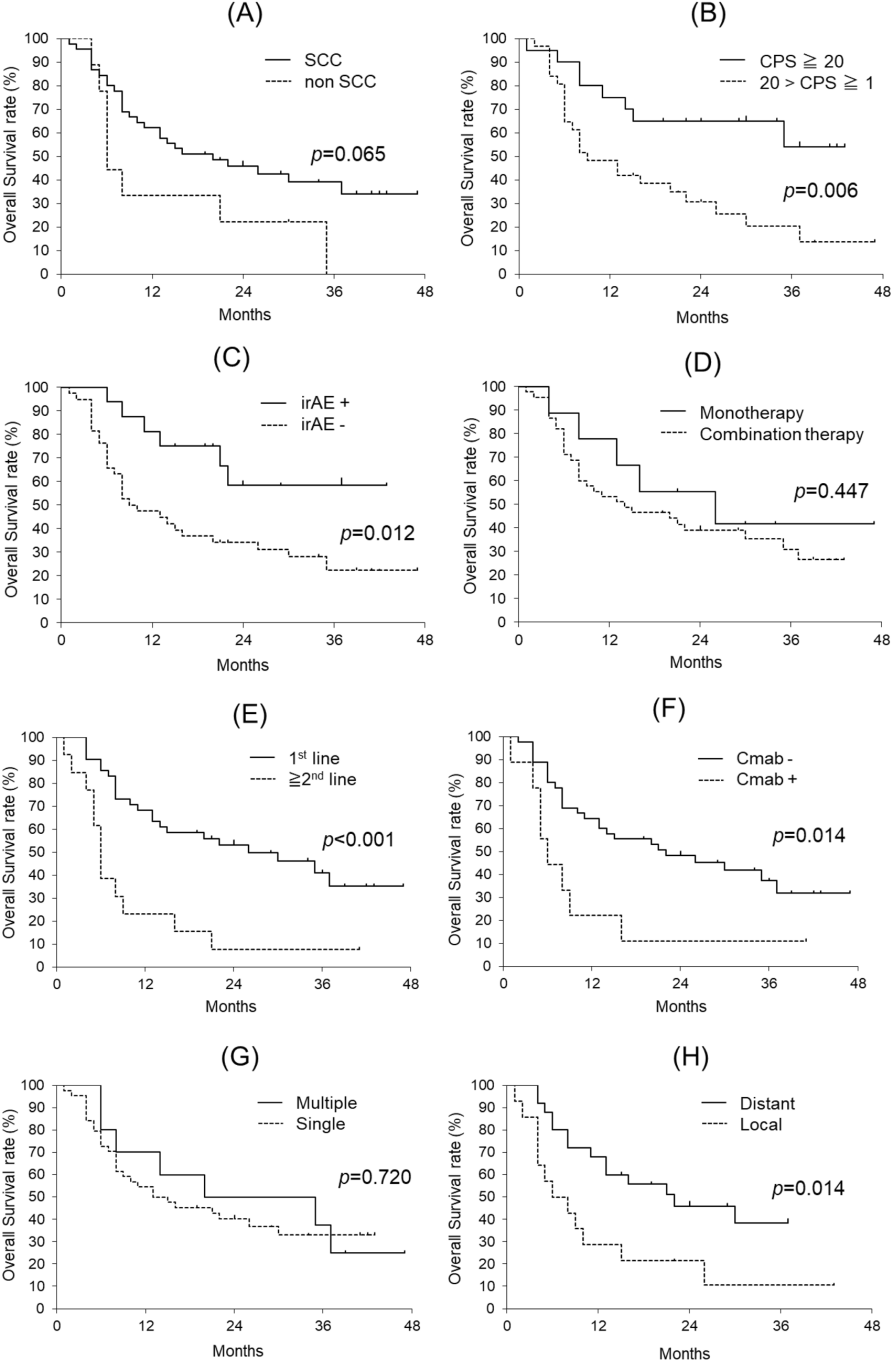
Overall survival curve and comparison by various subgroups. Squamous cell carcinoma (SCC) vs. other pathologies (non-SCC) (**A**), combined positive score (CPS) of 20 or higher vs. CPS 1–20 (**B**), with immune-related adverse events (irAEs) vs. without irAEs (**C**), pembrolizumab monotherapy vs. combination of pembrolizumab and chemotherapy (**D**), first line administration vs. administered second line or later (**E**), with a history of cetuximab (Cmab) treatment vs. without pretreatment of cetuximab (**F**), single-site recurrence vs. multiple site recurrence (**G**), and local recurrence vs, distant recurrence (**H**). SCC: squamous cell carcinoma

Figure 2A shows the survival curves comparing SCC (45 cases) and other histological types (nine cases). The 2-year OS rates were 45.9% and 22.2%, respectively, with SCC showing better outcomes; however, the difference was not statistically significant (p = 0.065). Figure 2B shows the survival curves based on the CPS differences. The 2-year OS rate for CPS 20 or higher (n = 20) and CPS 1–20 (n = 31) (3 cases were unmeasured) was 65.0% and 30.6%, respectively, with a statistically significant difference (p = 0.006). Figure 2C shows differences based on the presence or absence of irAEs. The 2-year OS rates were 58.3% in the irAE group (n = 13) and 34.2% in the non-irAE group (n = 38), with a statistically significant difference (p = 0.012). Figure 2D illustrates the differences between regimens. The 2-year OS rate was 39.0% for pembrolizumab monotherapy (n = 45) and 55.6% for combination therapy (n = 9), with no significant difference (p = 0.447). Figure 2E shows the differences in the pembrolizumab treatment sequence. The 2-year OS rate for cases in which it was used as first-line therapy (n = 41) was 53.1%, compared to 7.7% for those used as second-line or later treatment (n = 13), showing a statistically significant difference (p < 0.001). Figure 2F illustrates the differences in prior cetuximab administrations. The 2-year OS rate was 11.1% for those with a history of cetuximab administration (n = 9) and 48.2% for those without (n = 45), with a significant difference (p = 0.014). Regarding the number of recurrent lesions, comparing single-site recurrence (n = 44) and multiple-site recurrence (n = 10), the 2-year OS rates were 40.1% and 50.0%, respectively, with no significant difference (p = 0.720) (Fig. 2G). For cases with a single lesion, comparing local recurrence (n = 14) and distant recurrence (n = 25), the 2-year OS rates were 21.4% and 48.7%, respectively, with a statistically significant difference (p = 0.014) (Fig. 2H).

Upon conducting univariate analysis on the above factors, a significantly better prognosis was observed in cases with CPS 20 or higher (p = 0.008), cases that presented with irAE (p = 0.022), first-line administration cases (p = 0.003), and cases without a history of cetuximab administration (p = 0.030). Further multivariate analysis of these factors revealed significant differences for CPS 20 or higher (p = 0.030) and first-line administration (p = 0.038) (Table 3).

**Table 3:**
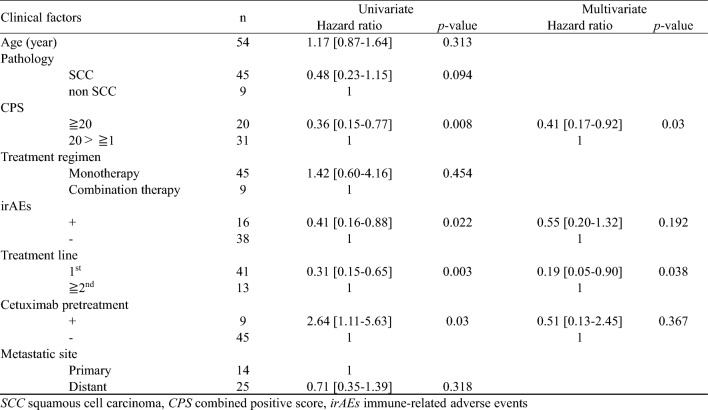
Univariate and multivariate analysis of the clinical factors associated with overall survival

### Discontinued cases

Although they achieved PR/CR once, there were 6 cases in which pembrolizumab administration was discontinued for reasons other than PD. The details are presented in Table 4. Five patients had hypopharyngeal cancer, and one had sinonasal cancer, all of which were SCC. All patients were male, aged between 52 and 78 years, with a median age of 72.5 years. In all cases, pembrolizumab was used as the first-line therapy, and no new treatment was initiated after discontinuation. The reasons for discontinuation were irAEs in three cases, patient requests in two cases, and one case where treatment could not be continued due to social reasons. Of the six patients, four maintained response after discontinuation, while two showed PD. Both PD cases had a high CPS of 90 or higher, whereas some cases that maintained response had a CPS below 20. When the treatment periods of the cases in this study were calculated based on the amount of pembrolizumab administered, the four patients maintaining tumor reduction had treatment durations of 78, 42, 36, and 36 weeks, while the two patients with PD had durations of 24 and 12 weeks, respectively. There was a tendency for patients who had longer treatment durations to maintain response. No correlation was found with other factors, such as the site of recurrence or the presence of irAEs.

**Table 4:**
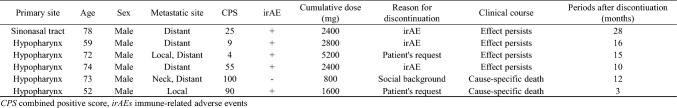
Characteristic of patients who discontinued Pembrolizumab

## Discussion

In this study, univariate analysis identified the CPS, sequence of pembrolizumab administration, presence of irAEs, and prior cetuximab treatment as factors significantly associated with survival. The first two factors were confirmed using multivariate analysis.

The CPS is used clinically as a predictor of ICI efficacy. ICIs have shown effectiveness against various types of cancer; however, the threshold for the CPS or tumor proportion score (TPS) is set based on clinical trial results and varies depending on the type of cancer. For R/M HNSCC, the CPS threshold is set at 1 or above (Chow et al. 2016); for advanced esophageal cancer, it is set at 10 (Sun et al. 2021); and for first-line lung cancer, effectiveness is seen at TPS 50 or higher (Paz-Ares et al. 2018). Since ICIs are antibody drugs, it is expected that a stronger antigen–antibody reaction would occur with a higher CPS, but clinical trials have not directly compared the effects based on CPS. This study represents the first report that directly compares groups with CPS ≥ 20 and CPS between 1 and 20, which were the thresholds used in the KEYNOTE-048 trial (Burtness et al. 2019), and the findings demonstrated a significant difference in survival rates. Recent reports on the efficacy of pembrolizumab for HNC indicate that, in a study of 139 cases, the factors correlated with survival rate were irAEs and performance status (PS), with no significant difference found when comparing CPS across three groups: CPS ≥ 20, CPS 1–20, and CPS < 1 (Matsuo et al. 2023). Similarly, in another study involving 66 cases, although significant differences in survival rates were observed based on PS, the presence of distant metastasis, and tumor shrinkage rate, no significant differences were found when CPS was examined in two groups: CPS ≥ 1 vs. < 1 and CPS ≥ 20 vs. < 20 (Saijo et al. 2023). In contrast, Saito et al. (Saito et al. 2024), in their study of 51 cases, reported a significant difference in the CPS and serum albumin levels. Although no difference was observed using a CPS cutoff of 20 (as in our study), significant differences were noted when comparing CPS ≥ 90 versus CPS < 90. There are reports that, with nivolumab, cases with PD-L1 expression of 40% or higher had better prognoses than those with less than 40% (Okamoto et al. 2020). In cancers other than HNC, lung cancer patients with a TPS > 90 showed higher effectiveness than those with a TPS < 90 (Edahiro et al. 2019). In other words, higher CPS (or TPS) values may suggest greater efficacy of ICI administration. In this report, among the 20 cases with a CPS of 20 or higher, 16 had a CPS of 50 or higher, and seven had a CPS of 90 or higher, suggesting that the relatively high CPS in many cases may have influenced the results.

There is a question of whether the effect persists even after discontinuation of pembrolizumab in cases where it has been effective. Considering the patient’s burden and medical costs, it is desirable to discontinue treatment if tumor shrinkage can be maintained. Currently, there are no established criteria for discontinuing treatment, and it is generally common to continue pembrolizumab administration even if CR is maintained. In this study, we examined six patients for whom treatment was discontinued after CR for various reasons. In three cases, treatment could not be continued due to irAEs; in two cases, treatment was discontinued at the patient’s request; and in one case, the decision was made due to financial reasons and difficulty in attending outpatient visits. Of these, four patients maintained response, while the remaining two patients showed PD after discontinuation of treatment. Upon examining the CPS, presence or absence of irAEs, sites of recurrence, and dose of pembrolizumab, it was observed that higher doses of pembrolizumab tended to be associated with maintained tumor reduction. Regarding CPS, the two patients with PD had high scores of 100 and 90, whereas those maintaining tumor reduction had scores of 55, 25, 9, and 4, with half of them being under 20. Although this was a small-scale study, the results suggest that the CPS may not be a reliable criterion for deciding when to discontinue treatment. To date, no studies have examined whether pembrolizumab should be discontinued in patients with HNC. In lung cancer, there has been a report comparing groups that discontinued nivolumab after one year with those who continued treatment, showing better OS and PFS in the group that continued treatment (Waterhouse et al. 2020). However, in another study comparing groups that discontinued ICIs after two years with those that continued ICIs, there was no difference in OS, suggesting that discontinuation could be considered for cases maintaining tumor reduction for two years (Sun et al. 2023). Both studies focused on the duration of ICI administration; however, in the present study, cases with shorter treatment periods resulted in PD. It is possible that even in HNC, the duration of pembrolizumab administration may influence the persistence of its effects after discontinuation. In this study, a response was maintained in cases with a longer duration of pembrolizumab administration. However, based on reports from lung cancer studies, the cases maintaining a response in this study still had relatively short follow-up periods, and continued observation is warranted. Since the number of cases was small and the observation period was short, further follow-up and analysis of more cases are necessary to evaluate the feasibility of discontinuing pembrolizumab.

This study had several limitations. First, this was a retrospective analysis conducted at a single institution with a limited number of cases. Further analysis with a larger sample size is warranted. Second, the choice between pembrolizumab monotherapy and combination therapy with chemotherapy was determined by considering the patient’s general condition. Therefore, selection bias might have influenced the results. Third, the observation period was short. Particularly, for cases in which pembrolizumab treatment was discontinued, the observation period was insufficient, and caution is needed when interpreting the results. Another point to note is the inconsistency in the timing and methods of measuring the CPS in this study. CPS is considered a dynamic value that can fluctuate due to various factors, such as tumor cell expression, immune cell infiltration (Fu et al. 2022), and some treatment (Karabajakian et al. 2021). Some reports suggest that whenever possible, multiple biopsies should be taken from the same tumor to evaluate CPS, rather than relying on a single specimen and that it is useful to repeatedly measure CPS during treatment (Meliante et al. 2023). In this study, CPS was evaluated using specimens collected from the recurrence site before the start of treatment. However, when sampling was not feasible, surgical or biopsy specimens obtained during the initial treatment were evaluated.

## Conclusion

In this study, cases of HNC with a CPS of 20 or higher showed a significantly better prognosis and were considered suitable for pembrolizumab administration. Furthermore, since favorable outcomes were observed in cases where pembrolizumab was used as initial therapy, it should be considered as a first-line treatment.

## Data Availability

No datasets were generated or analysed during the current study. The datasets generated and analysed in the current study are available from the corresponding author on reasonable request.
